# Effectiveness of an educational and physical program in reducing accompanying symptoms in subjects with head and neck pain: a workplace controlled trial

**DOI:** 10.1007/s10194-011-0291-y

**Published:** 2011-01-20

**Authors:** Eugenia Rota, Andrea Evangelista, Giovannino Ciccone, Luca Ferrero, Alessandro Ugolini, Chantal Milani, Manuela Ceccarelli, Claudia Galassi, Franco Mongini

**Affiliations:** 1 Unit of Headache-Facial Pain, Department of Clinical Pathophysiology, University of Turin, 14 Corso Dogliotti, 10126 Turin, Italy; 2 Unit of Cancer Epidemiology (CPO-Piemonte), AOU S. Giovanni Battista, Turin, Italy

**Keywords:** Accompanying symptoms, Educational program, Physical program, Head pain

## Abstract

The objective of this study is to evaluate the effectiveness of an educational and physical program in reducing behavioral or somatic symptoms along with headache, neck and shoulder pain in a working community. A controlled, non-randomized trial was carried out in a working community and 384 employees were enrolled and divided into a study group (Group 1) and a control group (Group 2). The Group 1 received a physical and educational intervention, consisting of relaxation and posture exercises and the use of visual feedback. After 6 months, the intervention was administered to the Group 2. Both groups were then followed for an additional 6 months until the end of the trial. The presence of accompanying symptoms was investigated with a semi-structured interview using a checklist of 20 items, along with headache, neck, and shoulder pain parameters and the prevalence of generalized anxiety disorder and depression, in three clinical examinations at baseline, after 6 months and after 12 months. For each symptom, as well as the presence of any type of symptom, the differences between groups in the prevalence at the clinical examinations following the baseline were evaluated by applying logistic models. After 6 months, the probability of the presence of any type of symptom was significantly lower in the Group 1 (OR 0.69, 95% CI 0.56–0.85) with respect to the Group 2. After 12 months, the pooled estimation did not show any significant difference of symptom prevalence between groups (OR 0.80, 95% CI 0.64–1.00). In conclusion, this is the first longitudinal study relative to accompanying symptoms. Its results suggest the effectiveness of this cognitive program in reducing the burden of physical and psychiatric complaints in a large, working population.

## Introduction

It is well known that physical and psychiatric complaints are highly prevalent in the general population, are frequently chronic, and are often associated with an increased likelihood of psychiatric disorders [1].

In particular, patients suffering from headaches usually complain of numerous accompanying symptoms, both behavioral and somatic, the prevalence of which has been found to be higher as compared to normal controls [2].

The association between headache, mainly migraine, and several psychiatric disorders, most commonly anxiety and depression, has been extensively explored by epidemiological and prospective studies [3–7]. Subjects suffering from migraine are from 2.2 to 4.0 times more likely to develop depression. The relationship between headache and depression or anxiety seems to be bi-directional, with each disorder increasing the risk of the other disorder [8]. Considering this evidence and the disability related to headache, above all if chronic, a new scale, Italian Perceived Disability Scale (IPDS), has been recently proposed to be used both in basic research and in clinical practice when screening for comorbidity with emotional distress and disorders [9].

The accompanying symptoms were demonstrated to be more common in patients with chronic headaches (definition includes chronic migraine, “transformed” migraine, migraine with tension-type headache, if >15 headache days per month, and new daily persistent headache), patients with a higher frequency of severe headaches, and patients with depression or anxiety [10].

Furthermore, psychiatric comorbidity, in particular anxiety and mood disorders (Axis I, DSM-IV), was shown to be more strictly associated than headache type or chronicity, with an increased burden of accompanying symptoms in headache sufferers [2].

However, the natural history of accompanying symptoms in relation to that of headache has not yet been explored, as well as the effects of physical and cognitive treatments on this complex interplay of head pain, psychiatric comorbidity and psychosomatic symptoms.

The efficacy of non-invasive physical management in reducing the frequency of various forms of headache and neck pain has been previously assessed by several studies, but conflicting results have been reported [11–13]. However, the weight of the evidence was still limited and the majority of studies employed a too short follow-up period to achieve data on the persistent effectiveness of physical management. We have for sometime been applying a simple educational and physical programme designed to decrease muscle tension in the head, neck and shoulder area. This programme is so simple that patients can follow it on their own after a short initial instruction, and its cost is negligible. In our clinical experience, it reduced the frequency and intensity of headache and neck and shoulder area pain in a considerable number of patients. Based on this encouraging results, we recently carried out a controlled trial [14, 15], which demonstrated the effectiveness of this educational and physical programs in reducing headache and neck and shoulder pain in a working community (a large sample of employees in Turin, Italy). A significant decrease of about 40% of the monthly frequency of headache and neck and shoulder pain was observed in the study group (192 central registry office employees) compared to controls (192 peripheral registry office and central tax office employees) in the first 8 months of the study. Moreover, the index of headache or neck and shoulder pain, as well as the frequency of drug intake, decreased significantly in the treatment group [14]. Furthermore, the long-term (14 months from the beginning of the study) benefit of such a program in the intervention arm of the study (192 office employees) was also demonstrated [15].

In the same study sample, the presence of accompanying symptoms was assessed in each clinical examination, along with headache, neck and shoulder pain parameters.

The purpose of the present study is to investigate the variation in the prevalence of accompanying symptoms in the study group and in controls during the aforementioned controlled trial and to evaluate the effectiveness of the educational and physical program in reducing symptoms that accompany headache, neck and shoulder pain.

## Methods

### Study design and participants

**Fig. 1:**
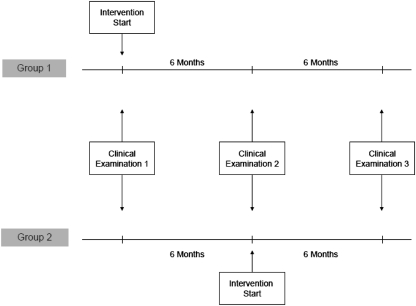
Flow chart

The design of this controlled, non-randomized trial has been extensively described in previous reports [14, 15] and is summarized in the flowchart (Fig. 1). The protocol was assessed and approved by the Institutional Review Board of the San Giovanni Battista Hospital of the city of Turin. Eligible participants included 661 employees of the City of Turin’s registry and tax offices as of 1 January 2005. Specifically, participants included 330 subjects from the central registry office (Group 1) and 331 at the peripheral registry offices and the tax office (Group 2). Informed consent was given by 192 and 192 subjects, respectively. No exclusion criteria were applied. Participants recorded daily pain episodes in diaries. After a 2-month period (March and April 2005), a first clinical examination (clinical examination 1, baseline) was carried out, and Group 1 received a physical and educational intervention consisting of a relaxation exercise performed once or twice a day, three posture exercises performed briefly every 2–3 h, and the use of visual feedback to monitor excessive contraction of the head and neck muscles. These instructions were reiterated to Group 1 again after 2 and 4 months. After 6 months (the eighth month since the beginning of the study), a second clinical examination (clinical examination 2) was performed for all subjects. The same physical and educational program was then administered to Group 2. Both groups continued to perform the exercises and fill out their diaries for months 9–14. A final clinical examination (clinical examination 3) was performed at the end of the 14th month of follow-up. A small number of participants were lost at the follow-up in both groups (35 drop-outs in Group 1, 28 in Group 2).

### Intervention

Before administering the program, an explanation was given concerning its main purposes, and particular emphasis was placed on the fact that some muscles, especially in the cranio-facial-cervical area, may be unconsciously maintained at too high a stage of contracture and that this may facilitate or increase pain in that area. Thus, the aim of the program was to reduce muscle contraction and to increase the capability of perceiving it when it is too elevated. The program consists of brief shoulder and neck (posture) exercises, a relaxation exercise, and instructions on how to reduce parafunction and hyperfunction of the craniofacial and neck muscles.

The posture exercises should have been performed 8–10 times, and repeated every 2–3 h, both in groups and individually. The characteristics of our programme were such that a reciprocal reinforcement of its educational aspects may be induced by more enthusiastic and collaborative participating subjects. When the instructions were reiterated at months 4 and 6 of the study period, these subjects were congratulated and encouraged to carry on with their commitment.

#### Posture exercises (8–10 times every 2–3 h)

(1) Stand upright with your heels, hips and nape of the neck against a wall. Without moving the rest of your body, bring your shoulders into contact with the wall and release rhythmically. (2) Stand with your body and head against the wall, make horizontal movements of the head, forwards and backwards, stretching out your head forwards and then retracting it against the wall. (3) Cup your hands behind your neck. Stretch and extend your head upwards and backwards against counter pressure from your hands. Relax forward after 2–3 s.

#### Relaxation exercise (once or twice a day, at home)

Sit down in a comfortable armchair in a quiet room. Let your lower jaw drop completely for about 10–15 min. Apply warm pads on your cheeks and shoulders.

#### Visual feedback

Place red labels in strategic sites to remind you to avoid excessive contraction of your head and neck muscles; when you see a red label, relax your face, neck and shoulders muscles.

Compliance during the entire study period was evaluated and classed as low (if exercises were performed no more than three/four times a week) and medium to high (if they were performed almost every day or exactly as indicated).

### Data collection

Detailed data related to headache and neck and shoulder pain were collected in a standardized fashion; a psychological assessment according to Axis 1 (Anxiety-, Mood- and Somatoform- Disorders) of the DSM-IV by means of a structured interview (SCID-I) [16] was conducted under the supervision of the same senior neurologist, and a clinical examination that included palpation of the pericranial and cervical muscles was performed by the researchers, whose interrating agreement was satisfactorily assessed in a blind fashion. Diagnoses of migraine with or without aura (M), tension-type headache (TTH), or myogenous neck and shoulder pain (MP) were made according to the guidelines of the International Classification of Headache Disorders [17] and the International Association for the Study of Pain [18]. Two or more diagnoses in the same subject were possible. All participants received a diary for daily self–reporting of severity (score 0–5) and duration (hours) of headache, and neck and shoulder pain, intake of analgesics (by type), and menstruation days. At the end of each month, diaries were collected, reviewed, and electronically processed by an optic reader and a dedicated computer program. All participants were asked to write in their diaries for the full 14 months.

The presence of accompanying symptoms, behavioral or somatic, was then investigated with a semi-structured interview, using a checklist of 20 items. In a previous study, these items [19] showed a significantly different prevalence when screened among healthy subjects and groups of patients suffering from hormonal, vascular, neurological and psychiatric disorders. Each symptom was considered positive if claimed as habitual or significantly present in the last 6 months, and it was recalled as annoying by the patient. We considered oral parafunctional habits such as tooth grinding, clenching, lip and nail biting, etc.

During each clinical examination, the aforementioned semi-structured interview, with the checklist of 20 items, was administered to all participants.

### Statistical methods

Baseline differences between Groups 1 and 2 were evaluated with the Mann–Whitney *U* test and the Chi-squared test for continuous or categorical variables, respectively.

According to the study design, statistical analyses concerning the study outcomes were performed to account for the repeated measurements framework. For each accompanying symptom, the differences between groups in the prevalence at the clinical examinations following the baseline were evaluated by applying logistic models. The presence of symptoms (yes/no) was the dependent variable. The group variable (Groups 1 and 2), time (clinical examination 2 and clinical examination 3) and their interaction were included in the analysis as independent variables, also adjusting for sex, age at enrolment and presence of the same symptom at baseline (which is assumed to be coincidental with clinical examination 1). In the same way, a pooled analysis, including the whole group of symptoms without distinction by type, was performed. In all analyses, pooled and by symptom type, the standard errors of the regression coefficients were adjusted for the clustering due to repeated measures within the same subject with the Huber-White Sandwich Estimator [20]. The differences between groups with respect to prevalence of subjects with generalized anxiety disorder (GAD) and depression during the follow-up were evaluated by applying logistic models in the same way as described above for each accompanying symptom.

## Results

Baseline characteristics of 384 subjects (192 in Group 1, 192 in Group 2) included in this study are summarized in Table 1. Due to the non-randomized design of the study, the two groups were not completely balanced with regard to age (subjects in Group 1 were significantly older than the subject of Group 2), and the prevalence of tension-type headache and myogenous neck and shoulder pain was higher in the control group.

**Table 1:** Characteristics of study population at the baseline (clinical examination 1)

	Group 1 (*n* = 192)	Group 2 (*n* = 192)	*p*
Age, median (IQR)	48 (43–53)	44 (36–50)	<0.001
Female, *n* (%)	150 (78.1%)	158 (82.3%)	0.305
Migraine with or without aura (M), without TTH, *n* (%)	53 (27.6%)	58 (30.2%)	0.574
Tension-type headache (TTH), without M, *n* (%)	33 (17.2%)	52 (27.1%)	0.020
Migraine and TTH, *n* (%)	51 (26.6%)	44 (22.9%)	0.408
Myogenous neck and shoulder pain (MP), *n* (%)	123 (64.1%)	146 (76.0%)	0.010

The results related to the frequency and intensity of headache or neck and shoulder pain and to analgesic drug consumption have been extensively described in previous reports [14, 15].

The prevalence of accompanying symptoms of GAD and depression at each clinical examination in both groups is reported in Table 2. A decrease of the prevalence of some symptoms (phobias, urinary disorders, etc.) may be observed after the intervention in both groups (in Group 1 at clinical examination 2 and in Group 2 at clinical examination 3).

**Table 2:** Symptom frequencies (%) by group at each clinical examination

	Group 1 Examination			Group 2 Examination		
	1 (*N* = 192)	2 (*N* = 169)	3 (*N* = 157)	1 (*N* = 192)	2 (*N* = 175)	3 (*N* = 164)
Symptom						
Colitis	21.4	21.9	21.0	27.6	21.7	23.8
Gastritis	19.8	20.1	19.7	18.2	22.3	18.3
Swallowing diff.	7.8	7.7	5.7	5.7	6.9	4.9
Digestion diff.	21.9	23.7	21.0	18.8	24.6	23.2
Phobias	18.2	9.5	10.2	15.6	14.3	9.8
Sleep disorders	46.4	37.3	39.5	39.1	41.1	41.5
Palpitations	24.5	19.5	15.9	25.0	29.7	28.0
Panic attacks	10.9	6.5	2.5	6.8	4.0	4.9
Fainting	1.6	1.2	1.3	5.7	3.4	0.0
Dizzines	20.3	21.9	16.6	18.2	18.9	18.9
Tinnitus	15.1	13.6	10.2	9.4	8.0	9.1
Weariness	35.9	39.6	52.2	42.7	45.1	53.0
Cramps	32.3	30.2	29.3	26.0	33.1	26.8
Paresthesias	33.3	26.6	28.7	28.1	32.6	29.3
Back pain	50.5	44.4	39.5	50.0	49.1	43.9
Urinary disorders	9.4	7.1	5.7	8.9	11.4	5.5
Circulation disorders	21.9	11.8	17.2	25.0	18.3	25.6
Anorexia/bulimia	19.3	16.0	17.2	11.5	11.4	15.9
Oral parafun.	27.6	39.6	33.1	32.8	39.4	33.5
Nail/hair fragil.	26.0	21.3	22.9	30.2	33.1	25.6
GAD	25.5	21.3	23.6	25.0	28.0	18.3
Depression	15.1	11.2	7.7	14.1	13.8	9.2

Examination 1 = baseline; examination 2 = after 6 months, when only Group 1 received the intervention; examination 3 = at the end of the study, when Group 2 also received the intervention

**Fig. 2:**
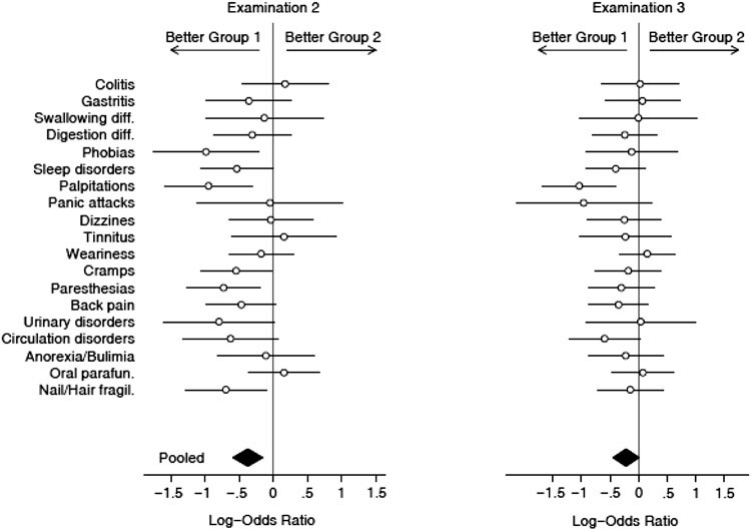
Differences between groups on the prevalence of accompanying symptoms (logistic regression models). The estimations are not plotted for fainting due to very large standard errors

Formal comparisons between groups of differences in the prevalence of symptoms, using logistic regression models, are shown in Fig. 2. At examination 2, Group 1 with respect to Group 2 showed a significantly lower probability of accompanying symptoms concerning phobias (OR 0.37, 95% CI 0.17–0.81), palpitations (OR 0.39, 95% CI 0.20–0.73), cramps (OR 0.58, 95% CI 0.35–0.98), paresthesias (OR 0.48, 95% CI 0.28–0.82) and nail/hair fragility (OR 0.50, 95% CI 0.28–0.90). Globally, considering the pooled estimation, the probability of any type of symptom was significantly lower in Group 1 than in Group 2 (OR 0.69, 95% CI 0.56–0.85).

Considering the subgroup of subjects with at least four headache episodes per month at the baseline (77 on Group 1, 91 on Group 2) and the whole group of symptoms, the improvement seemed to be more evident for patients that achieved at least a reduction of 50% of headache episodes (55% on Group 1, 19% on Group 2) (OR 0.66, 95% CI 0.47–0.95) with respect to other subjects (OR 1.13, 95% CI 0.89–1.43).

At examination 3 (at the end of the study, when Group 2 had also received the intervention), only palpitation symptoms remained significantly lower in Group 1 with respect to Group 2 (OR 0.35, 95% CI 0.19–0.67), and globally, considering the pooled estimation, there was no significant difference between groups regarding the prevalence of symptoms (OR 0.80, 95% CI 0.64–1.00).

Statistical analyses concerning GAD and depression prevalence showed no significant differences between groups during examination 2 or during examination 3. Nevertheless, a trend toward an improvement of GAD and depression after the intervention was detected in both groups (Table 2).

Finally, compliance was assessed with respect to the general effectiveness of the educational and physical program in decreasing headache and neck pain; as previously reported [14], at examination 2, no significant difference was detected between subjects with a medium–high level of compliance and those with a low level of compliance.

## Discussion

To the best of our knowledge, this is the first longitudinal study concerning accompanying symptoms. The results confirm the high prevalence of psychosomatic symptoms in the general population [1] and demonstrate that the administration of a simple educational and physical program can significantly decrease the psychosomatic complaints in a large working community.

This finding may be a consequence of the cognitive program, but it may be partially due to the beneficial effects of such programs on the headache, neck and shoulder pain in the study population. In fact, this program was demonstrated to reduce about 40% of the monthly frequency of headache, neck and shoulder pain in the study group, compared to the controls, and to significantly (about 50%) decrease the frequency of drug intake at both clinical examinations (after 8 and 14 months, respectively) [14, 15]. Moreover, the improvement of accompanying symptoms was significantly higher in those patients who achieved a reduction of at least 50% of headache episodes as compared to other subjects. Hence, the long-term benefit of such an educational and physical program on the burden of accompanying symptoms in this large sample of employees in the city of Turin seems to be in strict accordance with its general efficacy on the head-neck pain.

Although the decreased frequency of some symptoms, such as back pain, cramps, paresthesias, etc., may be somewhat related to the reduction of pain, the improvement of most symptoms, such as phobias, palpitations, may be less likely to be explained by this effect. A more wide and complex educational component of the program (including the approach to the problem and discussion of its major aspects, periodical instruction reinforcement, reinforcement by more motivated subjects of the working community, visual feedback, etc.) may underlie this beneficial influence on the psychosomatic symptoms through psychological mechanisms involving expectation and conscious anticipation [21, 22]. In particular, the expectation of a clinical benefit, which is equivalent to the expectation of a reward, may yield a placebo response by triggering reward mechanisms [23]. The characteristics of our program were such that the instructions were reiterated at months 4 and 6 of the study period, the subjects were congratulated and encouraged to carry on with their commitment, and a reciprocal reinforcement of its educational aspects was likely induced by more enthusiastic and collaborative participating subjects. Indeed, this aspect probably explains the few drop-outs compared with some other trials [24, 25] and a remarkable placebo response, which may have be responsible, at least to a certain extent, for the decreased frequency of some symptoms.

Furthermore, the finding that the program was effective in reducing headache and neck pain, as well as the burden of symptoms, also in subjects whose compliance was not optimal, is in agreement with a pivotal role of psychological mechanisms, in addition to the strictly physical ones (muscular relaxation).

Regarding the relationship between the presence of depression and/or GAD and the psychosomatic symptom time course during the trial, the statistical analysis did not reveal any significant differences in the prevalence of GAD and depression between groups at clinical examination 2 and 3; however, a trend toward an improvement was observed, possibly underlying, at least partially, the decrease of psychiatric complaints such as phobias, panic attacks, etc. This putative mechanism may also be hypothesized considering previous evidence that accompanying symptoms in headache sufferers are more strictly associated with psychiatric comorbidities than to headache type or chronicity [2].

Some methodological characteristics of our study should be taken into account. The main weakness of the trial design is the lack of a formal randomization: the subjects were assigned to Group 1 and Group 2 according to their place of work. In fact, both an individual and a cluster randomization were very difficult because of the features of our program, which involved encompassing exercises and visual feedback measures in the workplace, with a subsequent high level of interference and contamination between subjects working in the same environment as half of them were in a single, central department. The consequence of the lack of formal randomization is a somewhat unbalanced distribution at baseline of some characteristics between the two groups, mainly age; however, it should be noted that the frequency of most symptoms did not differ significantly at the first clinical examination.

In spite of these limits, the study has some characteristics that differentiate it from previous ones, concerning accompanying symptoms. First and perhaps most important is its longitudinal design; whereas psychosomatic symptoms were previously investigated in cross-sectional studies, they may have been influenced by undercurrent factors, such as seasonal variation, etc. In addition, the study sample size was large, the follow-up period was considerably long, and the number of drop-outs relatively small. Furthermore, this is one of the few studies in which a simple, self-administered physical exercise program can be performed without leaving the workplace and/or at home and without the intervention of paramedical staff.

In conclusion, the data show the effectiveness of our cognitive program in reducing the burden of physical and psychological complaints in a large, working population. Moreover, these findings indicate that the benefit is sustained for a considerable time through the whole intervention duration.
